# δ^13^C and Water Use Efficiency in the Glucose of Annual Pine Tree Rings as Ecological Indicators of the Forests in the Most Industrialized Part of Poland

**DOI:** 10.1007/s11270-016-2750-1

**Published:** 2016-02-01

**Authors:** Barbara M. Sensuła

**Affiliations:** Institute of Physics - Center for Science and Education, Silesian University of Technology, Konarskiego 22B, 44-100 Gliwice, Poland

**Keywords:** Carbon stable isotopes, iWUE, Atmospheric pollution, Glucose

## Abstract

In this study, stable carbon isotope ratios in the glucose samples were extracted from annual pine tree rings as bio-indicators of contemporary environmental changes in heavily urbanized areas. The sampling sites were located in close proximity to point source pollution emitters, such as a heat and power plant “Łaziska” and steelworks “Huta Katowice” in Silesia (Poland). The analysed samples covered the time span from 1975 to 2012 AD, the time period of the development of industrialization and the modernization in the industrial sector in Poland, similarly as in Eastern Europe. This modernization was connected with EU legislation and the implementation of restrictive governmental regulations on emissions. The carbon isotope discrimination has been proposed as a method for evaluating water use efficiency. The measurements of carbon isotopes were carried out using the continuous flow isotope ratio mass spectrometer coupled to the elemental analyser. The δ^13^C values were calibrated relative to the C-3 and C-5 international standards. Diffuse air pollution caused the variation in δ^13^C and iWUE (the ratio between CO_2_ assimilation and stomatal conductance) dependency on the type of emitter and some local effects of other human activities. In this study, the first results of water use efficiency in glucose are presented. In the period of time from 1975 to 2012, the water use efficiency values increased from 98 to 122 μmol/mol.

## Introduction

Most of the modernizations in different plants and the industrial sector in Eastern Europe are connected with EU legislation and the implementation of restrictive governmental regulations on emissions. According to the report of the European Environment (2013), exposure to air pollution has also been linked to low birth weights in babies, and also to asthma, heart disease and kidney damage. The fine toxic particles that are inhaled by humans and deposited in the lungs get to enter the bloodstream. In Poland, similarly as in most countries all over the world, the systematic long-term monitoring of air pollutants is generally restricted to rural point source regions in urban areas. Even for those areas, air pollution emissions were not continually monitored, and data is only available for the last decades. The pollution impacts human, plants and animal life and different ecosystem processes. Tree ring series that present long-term data can be used to analyse the ecosystem changes caused by human activities (e.g. Ferrio et al., 2003; McCarroll and Loader, 2004; Pazdur et al., 2007; Keeling et al., 2010; Rinne et al., 2010; Gagen et al., 2011; Battipaglia et al., 2013; Saurer et al., 2014; Sensuła et al. 2015a, 2015b; Sensuła 2015).

Scots pine (*Pinus silvestris* L.) is considered to be sensitive to the anthropogenic effect (Schweingruber, Schweingruber. F.H 1996; De Vries et al., 2000; Sensuła et al., 2011b; Sensuła and Pazdur, 2013a, 2013b; Pazdur et al. 2013, Saurer et al. 2014). Few studies have been successful at inferring long-term trends of point source air pollution involving different types of industrial production such as power plants, chemical plants, copper and metal smelters (Szychowska-Krąpiec and Wiśniowski, 1996; Wilczynski, 2006; Wagner and Wagner, 2006; Malik et al., 2012; Sensuła et al. 2015a, 2015b). But there is still a lack of a stable isotope fractionation analysis of trees growing in the contemporary forest in one of the most industrialized part of Europe—the southern part of Poland, where the reclamation of degraded landscapes is taking place in the post-industrial period of time. The combination of several independent indicators constitutes a powerful tool as an example in environmental research.

The analysis of diffuse air pollution signal recorded in the stable isotope composition of trees can show responses to environmental changes (e.g. (Schweingruber. F.H 1996; De Vries et al., 2000; Sensuła et al. 2011a; Sensuła and Pazdur, 2013a, 2013b; Pazdur et al. 2013). Several studies have used different stable isotopic compositions of the leaves as bio-indicators (e.g. Ehrelinger and Vogel, 1993; Gebauer et al. 1994; Emmett et al., 1998; Sensuła 2015) in the analysis of diffuse atmospheric pollution.

Human alterations of the carbon cycles have influenced the dynamics, biodiversity and functioning of many ecosystems and ecological processes (Martin et al., 1988; Vitousek et al., 1997; Marland 2008). An increase in air pollution, land use, to fossil fuel and biomass burning, climate changes can be responsible, among others, for differences in carbon isotopic fractionation (Farquhar and Lloyd, 1993; Saurer et al., 2004; Guerrieri et al. 2011; Choi et al., 2005; McCarroll et al., 2009; Pazdur et al., 2013; Sensuła and Pazdur, 2013b).

Since the 1970s, most paleoclimate studies have concentrated on the α-cellulose analysis as the dominant and most easily isolated wood component (e.g. Craig, 1954; Libby and Pandolfi, 1974; Leavitt and Long, 1982; Ehrelinger and Vogel, 1993; McCarroll and Loader, 2004; Sensuła et al., 2006; Pazdur et al., 2007; McCarroll et al., 2009; Rinne et al., 2010; Savard 2010). Cellulose ([C_6_H_10_O_5_]_n_, m.w. above 1.5 · 10^6^) is the major constituent of all plant materials. The large molecular size and insolubility make it difficult to precisely determine the chemical and physical properties of the intact cellulose polymer. Cellulose is a linear homopolymer built from β-1,4-linked glucose units (Gardner and Blackwell, 1974; Sjostrom, 1993), and glucose (C_6_H_12_O_6_) is one of the main products of photosynthesis, and this molecule participates in respiration.

Carbon isotope fractionation is highly correlated with the ratio of photosynthetic carbon assimilation to transpiration; therefore, carbon isotope fractionation is highly correlated with plant water use efficiency (Farquhar et al., 1989; Farquhar and Lloyd, 1993). Water is commonly the most limiting environmental factor for tree growth, and water may be limiting in urban environments where different factors, such as, among others, elevated temperatures, can combine to increase tree moisture stress (e.g. Cregg, 1993). The relationship between δ^13^C and plant water availability is not linear, showing a saturation trend as water availability increases (Warren and Adams 2000), and over a global survey of δ^13^C values on conifers, δ^13^C reached an asymptotic value once there is no water deficit, namely when the ratio between precipitation and evapotranspirative demand equalled unity. The reason for that general trend is that the main factor relating δ^13^C with water inputs is stomatal conductance, which is expected to reach its maximum in non-stressed plants. Under optimum water availability, no further increments in stomatal conductance, and thus on δ^13^C, would be expected (Lambers et al., 1998). The magnitude and spatial patterns of water fluxes passing through stomata in natural forests along Europe remain highly uncertain (Saurer et al., 2014).

Temporal and spatial variability of carbon concentration on both global and regional scales is a pre-requisite for a better understanding of the dynamics of the carbon cycle and its response to the ever-increasing human impact connected among others with industrial activity and housing energy (IPCC, IPCC 2001; Levin et al., 2010). This information can be also crucial, for example, for the reclamation of degraded landscapes in the post-industrial period of time. Spatial variability and temporal trends in water-use efficiency of European forests till 2000 AD has been studied in several tree species. Increased atmospheric CO_2_ concentration may stimulate plant growth, indirectly through reduced plant water consumption and hence slower soil moisture depletion and directly through enhanced photosynthesis (Morgan et al., 2004). Experimental results show that plants are able to increase their water-use efficiency (iWUE) as CO_2_ levels rise (Ehlelinger, Ehleringer 1990; Morison, 1993). It should be mentioned that Poland and some other Eastern European countries entered the European Union in 2004. The industrial sector was limited in Poland. Despite the fact that there are still many factories, the pollution connected with production processes is limited and restricted due to pro-ecological politics. However, other sources of pollution connected with housing energy exist.

The objectives of this study were to determine the following: (1) the pattern of variation of carbon stable isotope composition in glucose of pines growing along the industrialized part of Poland, (2) spatial variability and trends in intrinsic water-use efficiency and the ratio of photosynthesis to stomata conductance in contemporary forests located across the most industrialized and important part of Poland, where large parts of the population do not live in a healthy environment, according to current standards and (3) whether increasing atmospheric CO_2_ concentrations and changing climate increased iWUE (as detected by changes in δ^13^C) at the glucose level.

## Materials and Methods

### Site Description

Scots pine (*Pinus silvestris* L.) grows in the Silesia Upland, which is one of the most polluted and highly populated regions in Poland. To compare the nature of the air-pollution signal and its influence on isotopic fractionation of pine trees, three forests were selected as a study area, (i) two areas in the vicinity of industrial companies and (ii) a forest, located ca 100 km from the industrial source of pollution.

**Fig. 1:**
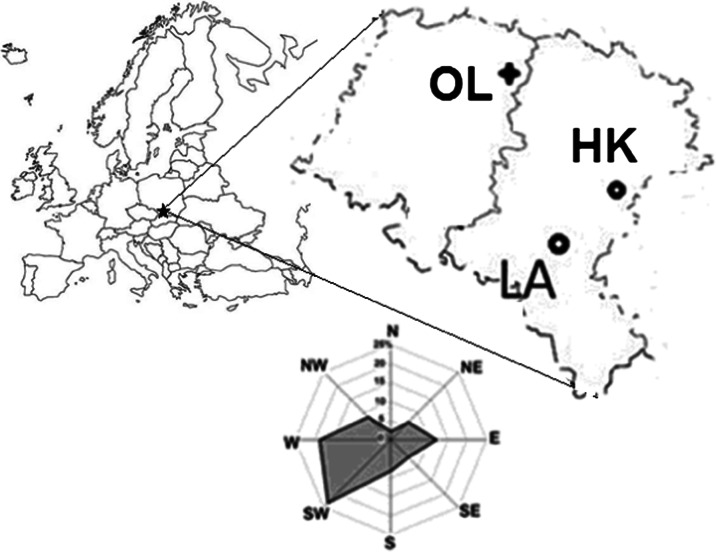
Sampling sites in the vicinity of the heat and power plant (*LA*) in Łaziska, near the Mittal Steel Poland steelworks (*HK*) and the comparative site (*OL*) ca 100 km NW from emitters

**Fig. 2:**
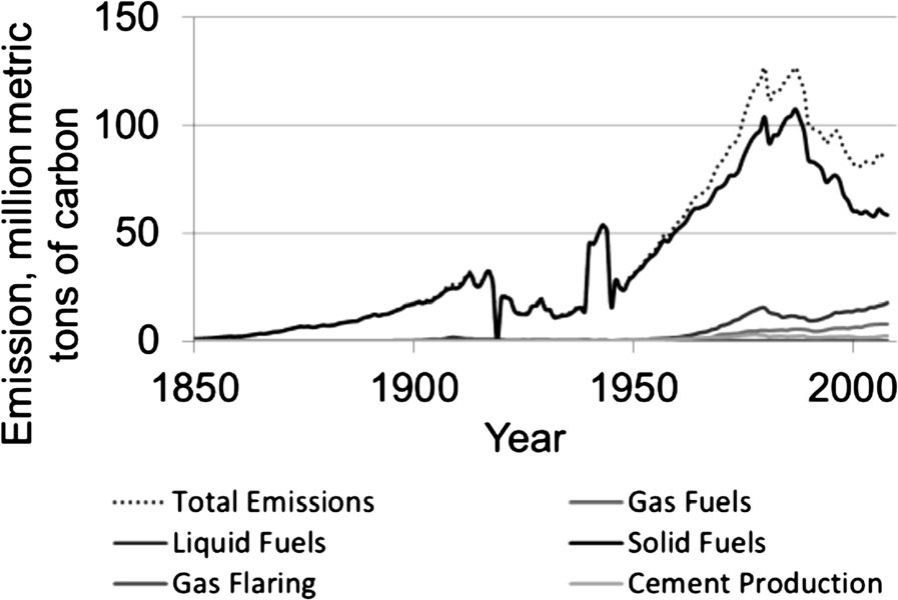
Emission of carbon in Poland from 1850 to 2010

The investigated sites were in the vicinity of the heat and power plant (LA) in Łaziska (50° 07′ 58″ N 18° 50′ 47.1″ E) and the Mittal Steel Poland steelworks (HK), former name: Huta Katowice, in Dabrowa Gornicza (50° 20′ 31″ N 19° 16′ 1″ E) and in one comparative site around 100 km NW from emitters (OL) (Fig. 1). Both factories have been on the list of the most environmentally burdensome industries in Poland for a long time. It should be mentioned that coal has been the traditional fuel for this region and has contributed 83 % of the total fossil fuel CO_2_ emissions in 1950. Most recently, the use of gas fuels has increased dramatically (Fig. 2), and since 1998, emissions from gas fuels have exceeded the emissions from coal (Marland 2008). According to a report of European Environment Agency (2013), a significant increase of pollution emissions is an effect of the industrialization of Silesia since the end of ninteenth century up to now in the investigated region. Most of the modernizations in those factories were connected with access to EU. In Poland, similar to the industrial sector in Eastern Europe, the implementation of restrictive governmental regulations on emissions was connected with EU legislation.

### Sampling and Dendrochronological Research

This article presents the investigations of carbon isotopic composition in samples of glucose hydrolysed from α-cellulose extracted from Scots pine annual tree rings (*P. silvestris* L.). Scots pine is the dominating tree species of the studied forests. This study is part of a large-scale research project (BIOPOL). Detailed dendrochronological investigations were part of the BIOPOL project, and the results have been described in detail in other publications (Sensuła et al., 2015a, 2015b; Sensuła, 2015). In the summer of 2013, more than 100 pine cores were collected at the breast height using a 5-mm diameter increment borer. In order to avoid a different dendroecological reaction of juvenile wood, an attempt was made to select pine stands aged between 90 and 100 years (felling age of Scots pine). The absolutely dated tree rings were manually separated as thin slivers and pooled and homogenized. The whole ring was sampled. The α-cellulose samples were extracted from increment cores of ten representative trees. The analysed samples covered the time span from 1975 to 2012 AD. The α-cellulose samples were extracted by applying the procedures based on the Green’s method (1963), which is a standard procedure used in the Mass Spectrometry Laboratory of the Silesian University of Technology (Sensuła et al. 2006; Pazdur et al., 2007, 2013; Sensuła et al. 2011b). The initial stage of the process was the sample milling into pieces of a size less than 0.5 mm and treatment with an ethanol-toluen mixture (in proportion 1:1 for 4 h at 80 °C), next with pure ethanol (for 4 h at 80 °C) and rinsing with distilled water (for 4 h at 100 °C) in Soxhlet extractor. The samples were dried overnight. The following step was bleaching with a NaClO_2_ and CH_3_COOH solution at 60 °C for 24 h in order to remove lignin and to extract cellulose. The resultant cellulose was rinsed with distilled boiling water, treated with 10 % NaOH for 1 h at 60° and treated with 17 % NaOH for 1 h at room temperature. The resultant cellulose was rinsed with distilled water. Afterwards, the samples were bleached with 2 % HCl for 15 min at room temperature. The resultant cellulose was rinsed with distilled water to a neutral pH. The samples were dried overnight at 50 °C. To ensure homogeneity and in order to obtain fine, uniform particle size for isotopic studies, the chemical pre-treatment was carried out in an ultrasonic bath. The hydrolysis of α-cellulose by 72 % H_2_SO_4_ was performed under the conditions described by Chambat et al. (1997). This method was adopted by the author in the Mass Spectrometry Laboratory of Silesian University of Technology in Poland (Sensuła et al. 2011b, Sensuła and Pazdur 2013a). Previous tests show that the precision of the isotope measurements in cellulose is less than in glucose. The precision on triplicates was ±0.3 ‰ (*n* = 50, for cellulose) and ±0.15 ‰ (*n* = 50, for glucose).

### Mass Spectrometry Analysis

The measurement of carbon isotopes was carried out using the continuous flow isotope ratio mass spectrometer coupled to the elemental analyser (EA-CF-IRMS). The isotope ratio mass spectrometers (IRMSs) are specialized mass spectrometers that produce precise and accurate measurements of variations in the natural isotopic abundance of light stable isotopes.

The stable carbon isotope compositions of the samples were determined using an IsoPrime elemental analyser/Continuous flow isotope ratio mass spectrometer (GV Instruments, Manchester, UK) at the Mass Spectrometry Laboratory of the Silesian University of Technology (Sensuła et al., 2011b, Sensuła and Pazdur 2013a, Sensuła and Pazdur 2013b). In order to determine the δ^13^C values, the samples (0.15 mg for carbon) were encapsulated in a tin. The samples were combusted at a temperature of 1020 °C in the elemental analyser. The δ^13^C values were calibrated relative to the C-3 and C-5 international standards. The δ^13^C results are reported in values relative to VPDB. The software Statistica 10 (Statsoft Inc. 2011) was used in all statistical analyses.

Isotopic values are reported in standard notation as delta:

1 $$ \updelta =\frac{R_{Sample}-{R}_{S \tan dard}}{R_{S \tan dard}} $$

where R represents the ratio of the heavy to light isotope in the sample and in the standard. The δ^13^C results are reported in values relative to VPDB.

The carbon isotope discrimination is calculated as follows (Farquhar and Lloyd, 1993):

2 $$ {\varDelta}^{13}C=\frac{\updelta^{13}{C}_{air}-{\updelta}^{13}{C}_{plant}}{1+\frac{\updelta^{13}{C}_{plant}}{1000}} $$

where ^13^C_air_ and δ^13^C_plant_ represent air and plant composition, respectively. The main factors determining δ^13^C in C_3_ plants are diffusion in the air (including the boundary layer and the stomata) and carbon fixation by the carboxylating enzyme ribulose bisphosphate carboxylase (Farquhar et al., 1989; Farquhar and Lloyd, 1993; Ferrio et al., 2003).

The isotopic discrimination in photosynthesis can be described as the model of (Farquhar et al. 1993):

3 $$ {\varDelta}^{13}C=a+\left(b-a\right){C}_i/{C}_a $$

where C_i_ is the intercellular CO_2_ concentration, C_a_ is the ambient CO_2_ concentration, *a* (ca. 4.4 ‰) is the discrimination against ^13^CO_2_ during CO_2_ diffusion through stomata, and b (ca. 27 ‰) is the discrimination associated with carboxylation.

4 $$ {\varDelta}^{13}C=a+\left(b-a\right)\left(1-1.6A/Ca\kern0.5em g{H}_2O\right), $$

where *A* is photosynthesis net, *g* is stomata conductance and 1.6 is the ratio of diffusivities of water and CO_2_ in the air.

5 $$ A=gC{O}_2\left({C}_a-{C}_i\right). $$

Carbon isotope discrimination (Δ^13^C) is related to intrinsic water-use efficiency (iWUE) which is the amount of water loss per unit carbon gained (Saurer et al., 2004).

6 $$ iWUE=A/g=\left({C}_a-{C}_i\right)/1.6. $$

### Meteorological Data

**Fig. 3:**
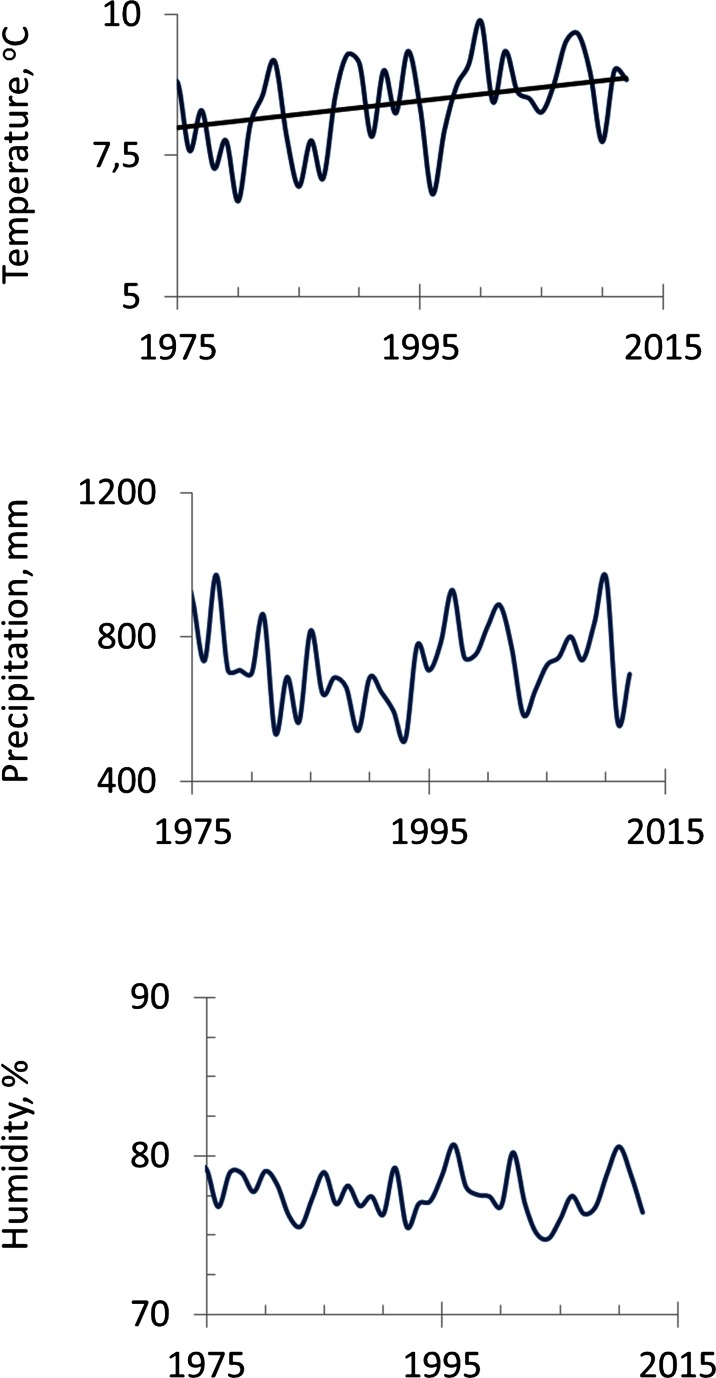
Climatic changes between 1975 and 2012 in Silesia (mean annual temperature, annual sum of precipitation and mean annual relative humidity). The rate of temperature increase is equal to 0.024 °C/year

The period from 1975 to 2012 AD is characterized in the regional climate records (the meteorological station in Katowice) by an annual mean temperature of approximately 8 °C (the rate of temperature increase is equal to 0.024 °C/year) and an annual sum of precipitation of approximately 743 mm (Fig. 3), and the dominant wind direction is from SE to NW (Fig. 1). The trend of the mean temperature is increasing. The vegetative period begins in April and lasts until September. The meteorological data were obtained thanks to the Polish Institute of Meteorology and Water Management (IMGW-PIB).

## Results and Discussion

**Fig. 4:**
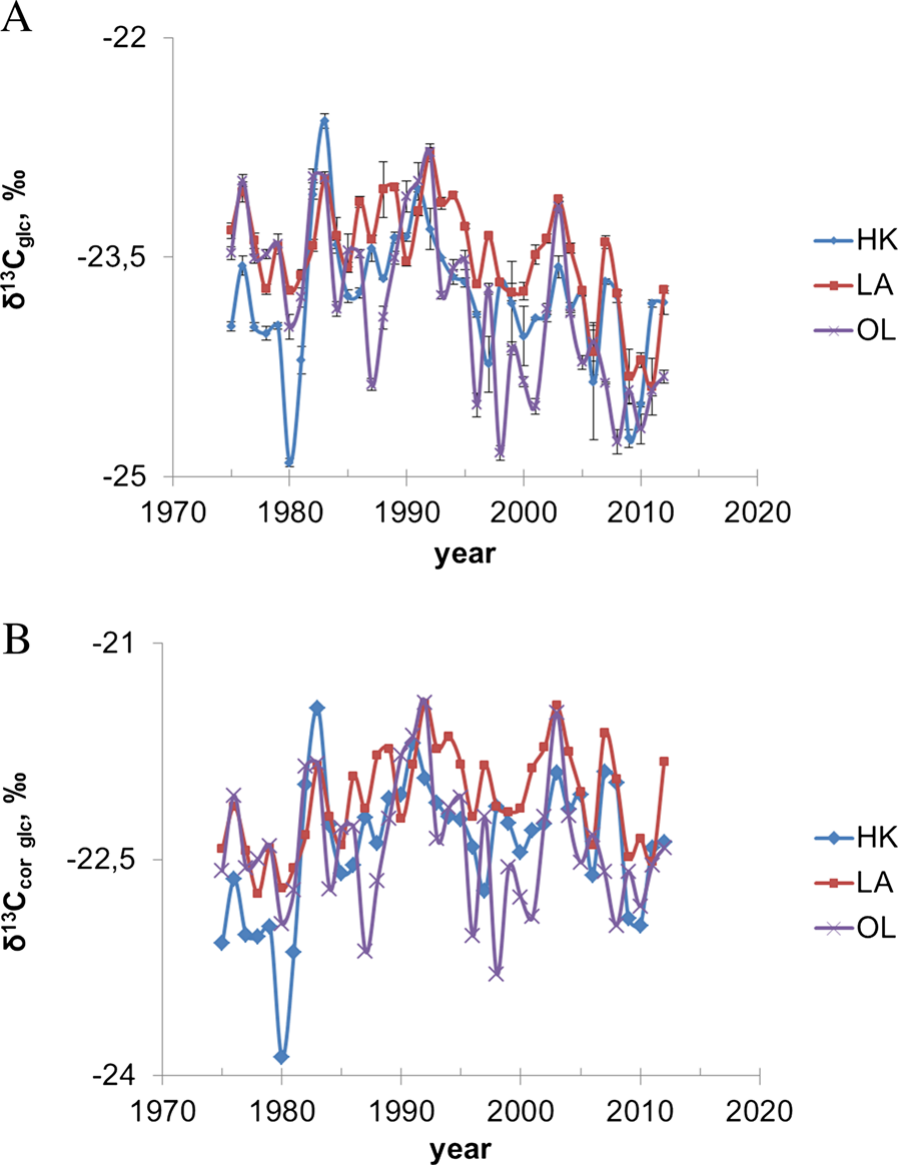
Variability of δ^13^C in glucose (**a**) raw value (**b**) isotopic data corrected for the depletion of global atmospheric δ^13^C due to fossil fuels burning

**Fig. 5:**
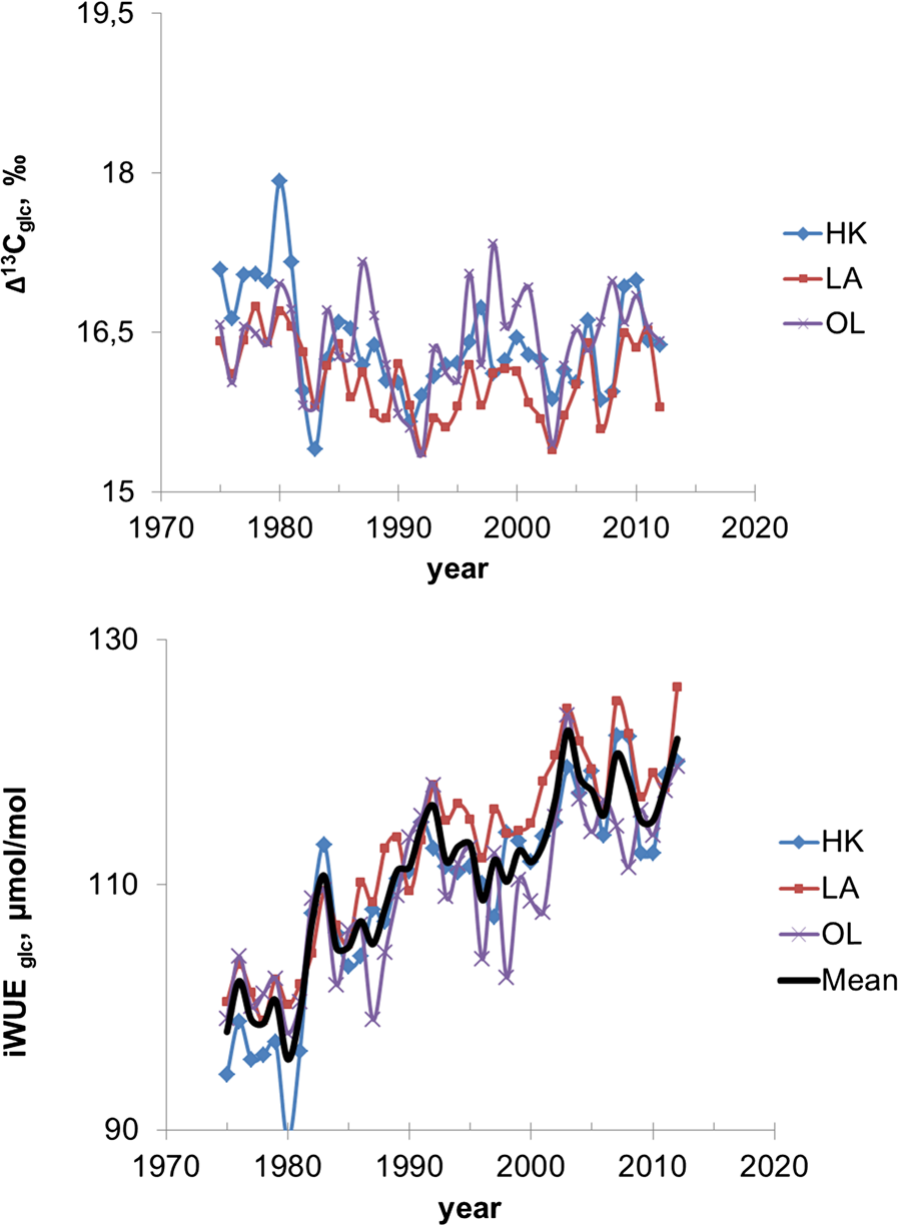
Variability of Δ^13^C and iWUE in Silesia (in vicinity of the heat and power plant (LA) in Łaziska, near the Mittal Steel Poland steelworks (HK) and the comparative site (OL) ca 100 km NW from emitters) in the period of time from 1975 to 2012

Pattern of spatial and short-temporal variability of δ^13^C, Δ^13^C and water use efficiency (iWUE) in glucose samples received from annual pine tree rings growing in three forests (in proximity to the heat and power plant in Łaziska (LA) and the steelworks in Dabrowa Gornicza (HK) and in the comparative site (OL) is illustrated (Figs. 4 and 5) and summarized in Table 1.

**Table 1:** δ^13^C, carbon isotope discrimination (Δ) and water-use efficiency values (minimum, maximum, average and standard deviation-SD) measured in glucose samples received from annual tree rings of pine growing in the area near the combined heat and power plant in Łaziska (LA), steelworks in Dąbrowa Górnicza (HK) and in the comparative site (OL)

	Site	Minimum	Maximum	Average	SD
δ^13^C, ‰	HK	−24.90	−22.57	−23.81	0.47
Δ^13^C, ‰	HK	15.40	17.92	16.39	0.49
iWUE, μmol/mol	HK	89.2	122.2	109.6	8.3
δ^13^C, ‰	LA	−24.38	−22.78	−23.48	0.37
Δ^13^C, ‰	LA	15.36	16.73	16.05	0.36
iWUE, μmol/mol	LA	98.8	126.1	112.8	7.5
δ^13^C, ‰	OL	−23.29	−21.41	−22.41	0.44
Δ^13^C, ‰	OL	15.36	17.33	16.40	0.46
iWUE, μmol/mol	OL	97.9	123.8	109.4	6.7

### δ^13^C Chronologies

δ^13^C values in glucose samples from pine growing in the forests located in proximity to industrial factories show a significant correlation between them (*r* = 0.72, *p* < 0.05), which confirm that similar trees response to the stress of industrial pollution emission. Whereas the correlation of δ^13^C between the industrialized area and the comparative site is much lower, in the case of HK and OL populations, it is equal to 0.42 (*p* < 0.05), and between LA and OL populations, it is equal to 0.45 (*p* < 0.05), respectively. The gradient changes of δ^13^C can be observed from year to year. Spatially inhomogeneous δ^13^C changes as a function of the type of source of pollution are illustrated (Fig. 4a). In general, higher value of δ^13^C is observed in proximity to the heat and power plant in Laziska. Raw δ^13^C values were corrected to a pre-industrial atmospheric δ^13^C base value of 6.4 ‰ using the data from McCarroll and Loader (2004). This correction lifts the δ^13^C values (Fig. 4b).

### iWUE Records

iWUE values in the forests in proximity to industrial factories show significant correlation between them (*r* = 0.92, *p* < 0.05), whereas the correlation of iWUE value between industrialized area and the comparative site is slightly lower, but also statistically significant (*p* < 0.05), and in the case of HK and OL populations, it is equal to 0.80, and between LA and OL populations, it is equal to 0.83, respectively. The gradient changes of iWUE can be observed from year to year. Spatially inhomogeneous iWUE changes as a function of the type of emitters can be noted (Fig. 5). In general, the highest value of iWUE is observed in proximity to the heat and power plant in Laziska (LA). The lowest values of iWUE are observed in the comparative site population of pine (OL). The iWUE inferred from the Δ^13^C analyses of trees increased 25 % over the last 40 years. A similar increasing trend could be observed in annual tree rings across Europe till 2000 AD (Saurer et al. 2014). It was found that the magnitude of tree response varied widely across sites and species (Warren et al. 2011) and appeared to be influenced by other environmental and growth-related factors (Leakey et al., 2009; Norby and Zak, 2011).

### Interaction with the Climate

**Fig. 6:**
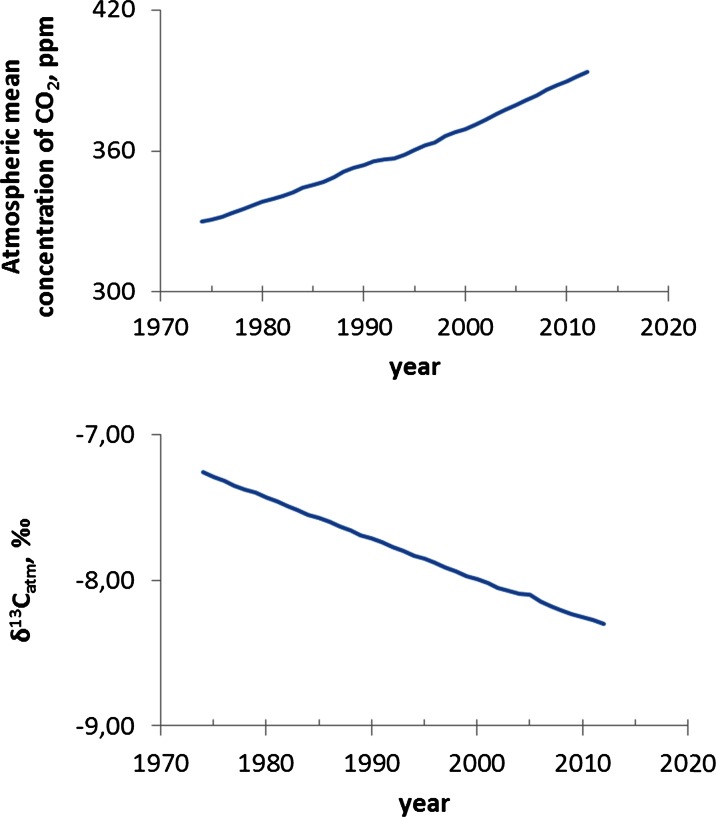
The relationship between the isotope time series and climatic records (significant level at *p* = 95 %)

The significant relationship (at the 95 % confidence limit) between the carbon isotope fractionation time series, carbon discrimination factor and iWUE and climatic records is illustrated (Fig. 6). A review of the literature shows that there has been much discussion about the impact of pollution on tree ring width, δ^13^C in cellulose and wood (e.g. Pazdur et al., 2007, 2013; Keeling et al., 2010; Gagen et al., 2011), but there is a lack of analysis of the impact of pollution on the climate signal recorded in the glucose molecule of pine growing in Eastern Europe during the development of industry and the reduction of pollution emission in the last decades. The previous analysis shows that in the period of time from 1950 to 2000 AD, the correlation coefficients between δ^13^C in glucose and the climate factors in the Niepolomice Forest (Poland) were not stable over the entire studied period (Sensuła et al., 2011b). Some changes in the mean monthly temperature, and the sum of precipitation does have an influence on the isotopic composition of glucose (Sensuła et al. 2009, 2011a, 2011b). The analysis of correlation from 1950 to 2000 based on the bootstrap correlation function shows a significant correlation between δ^13^C in glucose and August and May temperature as the most important climate signals recorded in the carbon isotope fractionation. The negative correlations with May temperature could also be due to the physiology of pine (such as dusting or seasonal variability of the level of auxin concentration in pine) (e.g. Sensuła et al. 2013). The influence of the winter temperature on tree ring’s radial growth has been observed in the area in proximity to factories (Sensuła et al. 2015). The carbon isotopic composition in plant varies with water stress (Ehleringer and Cooper, 1988) and solar radiation (Ehleringer et al., 1986) and has been correlated inter alia with rainfall amount (Stewart et al. 1995), vapor pressure deficit (Comstock and Ehleringer, 1992) and water use efficiency (Ehleringer et al. 1990).

The climatic signal recorded in stable isotopes can be masked and modified by air pollution. Only a few studies mention (i.e. Leonelli et al., 2012; Battipaglia et al., 2013; Sensuła et al. 2013b) the potential impact of air pollution on stable-isotope-based climate reconstructions. In the investigated forests, the pines of each population show a different sensitivity to weather conditions. The detailed relationship between the isotope time series and climatic records will be the subject of a further publication.

### HK Pine Population

The positive relationship with July (*r* = 0.53, *p* < 0.05) and August (*r* = 0.47) maximum temperature and negative relationship with August (*r* = −0.51, *p* < 0.05) and September (*r* = −0.41, *p* < 0.05) humidity are the most important climate signals recorded in the carbon isotope fractionation in glucose in the population growing in this industrialized site.

A high positive relationship with January (*r* = 0.45) and May (*r* = 0.39, *p* < 0.05) temperature shows that it is also an important climate signal recorded in the carbon isotope fractionation in glucose. The lower negative correlation is observed between δ^13^C and July humidity (*r* = −0.38, *p* < 0.05). The negative correlation is noted between precipitation in July (*r* = −0.33, *p* < 0.05) and August (*r* = −0.35, *p* < 0.05) and carbon isotope fractionation in glucose.

The inverse correlation is observed for carbon discrimination factor (Δ^13^C). The negative relationship with (*r* = −0.53, *p* < 0.05) and August (*r* = −0.47, *p* < 0.05) maximum temperature and the positive relationship with August (*r* = 0.51, *p* < 0.05) and September (*r* = 0.41) humidity are the most important climate signals recorded in the carbon isotope discrimination in glucose in the population growing in this industrialized site. The high negative relationship with (*r* = −0.45, *p* < 0.05) and May (*r* = −0.39, *p* < 0.05) temperature shows that it is also an important climate signal recorded in the carbon isotope discrimination in glucose. A lower positive correlation is observed between Δ^13^C and July (*r* = 0.38, *p* < 0.05) humidity. The positive correlation is noted between precipitation in (*r* = 0.33, *p* < 0.05) and August (*r* = 0.35, *p* < 0.05) and carbon isotope discrimination in glucose.

iWUE is correlated only with the relative humidity of the air and monthly maximum temperature. The positive relationship with July (*r* = 0.61, *p* < 0.05) and August (*r* = 0.48, *p* < 0.05) and May (*r* = 0.42) maximum temperature and the negative relationship with April (*r* = −0.53, *p* < 0.05) and August (*r* = −0.37, *p* < 0.05) humidity are the most important climate signals recorded in the carbon isotope fractionation in glucose in the population growing in this industrialized site.

### LA Pine Population

Some similar results are observed for the LA population as in the case of the HK population. The positive relationship with July (*r* = 0.49, *p* < 0.05) and August (*r* = 0.57, *p* < 0.05) maximum temperature and negative relationship with August (*r* = −0.60, *p* < 0.05) humidity are the most important climate signals recorded in the carbon isotope fractionation in glucose in the population growing in this industrialized area. The high positive relationship with January (*r* = 0.38, *p* < 0.05) and April (*r* = 0.33, *p* < 0.05) temperature shows that it is also an important climate signal recorded in the carbon isotope fractionation in glucose. The lower negative correlation is observed between July humidity (*r* = −0.35, *p* < 0.05), precipitation in August (*r* = −0.48, *p* < 0.05) and carbon isotope fractionation in glucose.

The inverse correlations are observed for carbon discrimination factor (Δ^13^C). The negative relationship with July (*r* = −0.49, *p* < 0.05) and August (*r* = −0.57, *p* < 0.05) maximum temperature and positive relationship with August (*r* = 0.60) humidity are the most important climate signals recorded in the carbon isotope discrimination in glucose in the population growing in this industrialized area. The high negative relationship with January (*r* = −0.38, *p* < 0.05) and April (*r* = −0.33, *p* < 0.05) temperature shows that it is also an important climate signal recorded in the carbon isotope discrimination in glucose. The lower positive correlation is observed between July humidity (*r* = 0.35, *p* < 0.05), precipitation in August (*r* = 0.48, *p* < 0.05) and carbon isotope discrimination in glucose.

iWUE is correlated only with humidity and maximum temperature. The positive relationship with July (*r* = 0.57, *p* < 0.05) and August (*r* = 0.51, *p* < 0.05) and June (*r* = 0.40, *p* < 0.05) maximum temperature and the negative relationship with April (*r* = −0.58, *p* < 0.05) and August (*r* = −0.36, *p* < 0.05) humidity and the positive relationship with January (*r* = 0.38) humidity are the most important climate signals recorded in the carbon isotope fractionation in glucose in the population growing in this industrialized site. Additionally, a significant correlation is observed between iWUE and temperature in April (*r* = 0.38, *p* < 0.05) and May (*r* = 0.36, *p* < 0.05).

### OL Pine Population

The negative relationship with August (*r* = −0.51, *p* < 0.05) and September (*r* = −0.50, *p* < 0.05) humidity is the most important climate signal recorded in the carbon isotope fractionation in glucose in population growing in the comparative site (OL). The lower negative correlation is observed between δ^13^C and July (*r* = −0.37, *p* < 0.05) humidity. The negative correlation is noted between precipitation in August (*r* = −0.40, *p* < 0.05) and carbon isotope fractionation in glucose. The positive relationship with August (*r* = 0.33, *p* < 0.05) maximum temperature is observed, whereas there is no significant correlation between January temperature.

The inverse correlations are observed for the carbon discrimination factor (Δ^13^C). The positive relationship with August (*r* = 0.51, *p* < 0.05) and September (*r* = 0.50, *p* < 0.05) humidity is the most important climate signal recorded in the carbon isotope discrimination in glucose in the population growing in the comparative site (OL). The lower positive correlation is observed between Δ^13^C and July (*r* = 0.37, *p* < 0.05) humidity. The positive correlation is noted between precipitation in August (*r* = 0.40, *p* < 0.05) and carbon isotope discrimination in glucose. The negative relationship with August (*r* = −0.33, *p* < 0.05) maximum temperature is observed, whereas there is no significant correlation between January temperature.

iWUE is correlated only with humidity and maximum temperature. The positive relationship with July (*r* = 0.55, *p* < 0.05) and August (*r* = 0.62, *p* < 0.05) maximum temperature and the negative relationship with April (*r* = −0.50, *p* < 0.05), August (*r* = −0.43, *p* < 0.05) and September (*r* = −039, *p* < 0.05) humidity and the positive relationship with January (*r* = 0.42, *p* < 0.05) humidity are the most important climate signals recorded in the carbon isotope fractionation in glucose in population growing in this site.

### CO_2_ Impact on δ^13^C and iWUE

These studies show that the relative importance of different sources of carbon for the plant carbon budget can be derived at glucose by using the C isotope (global emission of CO_2_ from fossil fuel combustion, local emission of pollution by industrial sector, low stack emissions of CO_2_ from homes and housing energy from home). A decrease of δ^13^C in glucose has been observed since 1975 (Fig. 4a). The effect of the decrease can be explained as the known effect of the decreasing δ^13^C in the air, and the biosphere is associated with the increase of CO_2_ in the atmosphere (Craig 1954; Polley et al., 1993; McCarroll and Loader, 2004; Pazdur et al., 2007, 2013; Keeling et al., 2010; Sensuła et al. 2013a, 2013b).

**Fig. 7:**
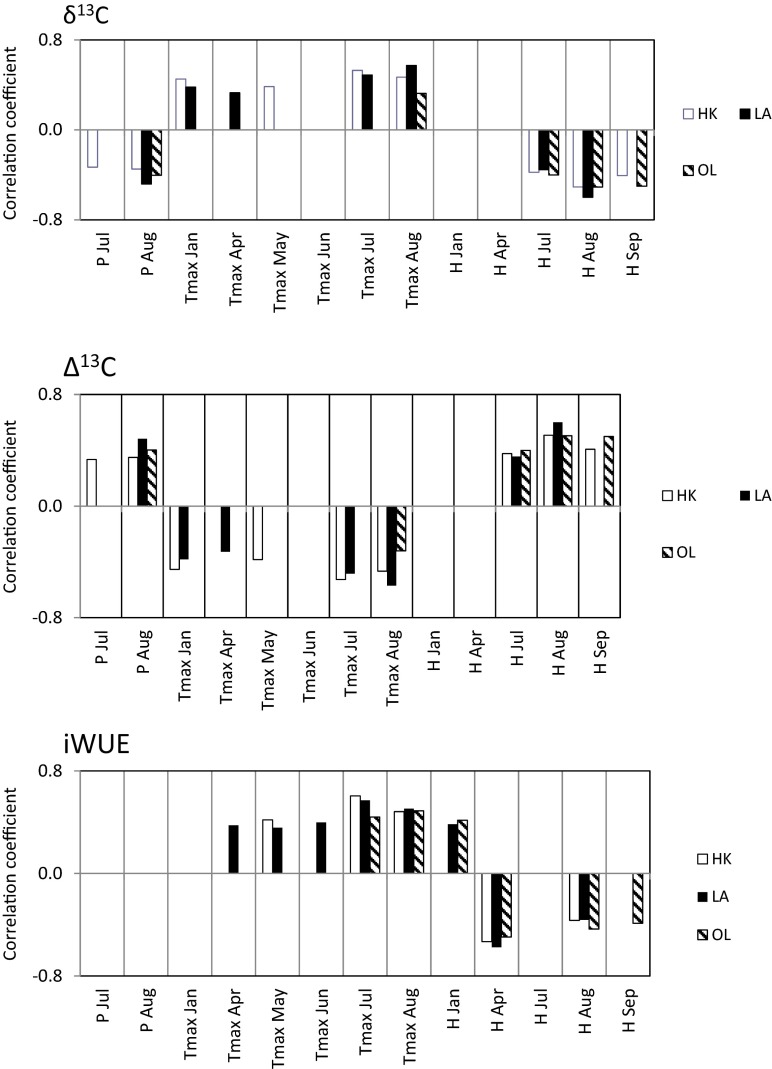
Elevation of global atmospheric CO_2_ concentration and decreases in δ^13^C in the period of time from 1975 to 2012 (according to NOAA-ESRL annual data posted at ftp://ftp.cmdl.noaa.gov/ccg/co2/trends/co2_annmean_mlo.txt)

According to the NOAA, the average global atmospheric CO_2_ concentration has risen from 331 ppm in 1975 to 393 ppm in 2012. The observed anthropogenic impact on the carbon cycle is mainly related to various industrial activities (Marland et al., 2008) that caused changes in the isotopic composition of carbon in the ecosystem (Pazdur et al., 2013). Plants grown at the higher level of CO_2_ concentration had a more negative δ^13^C than plants grown at the lower concentration. In pine, similarly as in C_3_ plants, CO_2_ usually limits photosynthesis, and, thus, an increase in CO_2_ results in greater photosynthetic rates. Elevated CO_2_ significantly decreases δ^13^C in the air. The δ^13^C present in the air is about −8.3 ‰ (Fig. 7).

Also, lower values of δ^13^C (before 1980) correspond to the highest levels of dust and gaseous pollutants, which were recorded in the southern part of Poland in the late 1970s (Marland et al. 2008).

Plants took advantage of the increased CO_2_ availability to augment water-use efficiency (i.e. the ratio between net assimilation and water transpired) by closing stomata. The relationship between δ^13^C and plant water availability is not linear (*r* = 0.76 for HK population, *r* = 0.68 for LA population, *r* = 0.56 for OL population, respectively), showing a saturation trend as water availability increases. The reason for that general trend is that the main factor relating δ^13^C with water inputs is stomata conductance, which is expected to reach its maximum in non-stressed plants (Warren et al. 2001). Variation in iWUE estimated as the ratio between photosynthesis and stomatal conductance is recorded in the variation of the carbon isotope discrimination Δ^13^C of the annual growth rings that are laid down during each growing season (e.g. Duquesnay et al., 1998). Elevated CO_2_ significantly increases the intrinsic water-use efficiency (iWUE) of forests, and the increase of iWUE values is observed with the elevation of global atmospheric CO_2_ concentration in all investigated sites. When plants are exposed to elevated CO_2_ levels, a decline in stomata conductance (*g*) can be predicted (Field et al., 1995). This reduction in stomatal conductance does not limit photosynthesis; thus, δ^13^C values become more negative as CO_2_ concentration increases (Polley et al., 1993). Therefore, CO_2_ and light gradients may have additive effects within closed canopies, both contributing to leaf δ^13^C decrease with depth (Broadmeadow and Griffiths, 1993).

### Other Pollution Effects

The influence of SO_2_ on δ^13^C values cannot be excluded. SO_2_ emissions can increase the tree ring δ^13^C values by augmenting dark respiration and changing the photosynthate allocation and partitioning (Wagner and Wagner, 2006; Rinne et al., 2010). There is a lack of SO_2_ pollution data for the investigated areas since 1975 (this is also due to changes in the administrative division of Poland and due to the lack of access to data from both factories, and lack of records for Olesno for the entire investigated time period). On the other hand, a depletion of δ^13^C values can be due to fossil fuel emissions connected with local road effect or other potential sources of pollution. The depletion of δ^13^C values in glucose samples of OL population shows that lower values of δ^13^C can be connected not only with the industrial emission of pollution. Olesno is located about 100 km NW from the heavily industrialized area of Silesia. According to the Regional Inspectorate for Environmental Protection (WIOS), recently, the air quality is deteriorating in Olesno from year to year, especially with the beginning of the heating season. In this region, depletion in δ^13^C is mainly connected with low stack emissions of CO_2_ from homes and housing energy because many people in Olesno did not only burn coal in furnaces but also plastic bottles and clothing in their homes. As a result, the air contains toxic components, among others, toxic dioxins, furans, heavy metals, benzene and nitrogen oxides, which influence human, animal and plant life. There is no national standard concerning the level of low lack stack emission in Poland. But it is known that low lack emission can cause allergies, asthma and cancer. The pollution has an influence on human, animal and plant life, and it can cause allergies, asthma and cancer.

## Conclusions

The various human activities, mainly related to fossil fuel burning and land use changes, have an impact on the carbon cycle and have caused changes in the isotopic composition of carbon in the atmosphere and in the biosphere.

In this study, it was demonstrated that the carbon isotope indicators can provide information on diffuse air pollution, and this is coherent with the investigated region. This approach can provide complementary information to reconstruct and analyse the environmental perturbations.

These studies confirm that the physiological responses (stable isotopic fractionation and variation of iWUE at the level of glucose) over the last decades were modified as a function of the type of emitters, distance from emitters and some local effect such as local road effects and low stack emissions. Low stack emissions can significantly influence δ^13^C and iWUE values. Low quality fuels, lack of standards for furnaces used in homes and the lack of awareness of the inhabitants and their difficult economic situation are the main reasons for the great troubles with low stack emissions in Poland and Eastern Europe. Elevated emission of CO_2_ and elevated temperatures increase iWUE. The increase of global atmospheric CO_2_ concentration to about 60 ppm increased by about 25 % iWUE over the last 40 years. The relation between δ^13^C and iWUE is not linear. δ^13^C and iWUE also depend on climate changes. Different responses to climate are noted by the pine population growing in the vicinity of both factories and the pine population growing in the comparative site. However, the reaction of trees growing in the vicinity of factories is similar. The most important climate signal recorded in the δ^13^C, Δ^13^C and iWUE, in general, is humidity and maximum temperature, especially humidity and maximum temperature in August. A high correlation between δ^13^C and temperature in January and a high correlation between humidity at the beginning of vegetation period in April and iWUE are interesting phenomena. The detailed relationship between the isotope time series and climatic records will be the subject of further research and publication.
